# Case Report: Successful DaVinci-Assisted Major Liver Resection for Alveolar Echinococcosis

**DOI:** 10.3389/fsurg.2021.639304

**Published:** 2021-03-04

**Authors:** Mohammad Golriz, Viktoria Flossmann, Ali Ramouz, Ali Majlesara, Yakup Kulu, Marija Stojkovic, Arianeb Mehrabi

**Affiliations:** ^1^Department of General, Visceral, and Transplantation Surgery, University of Heidelberg, Heidelberg, Germany; ^2^Department of Clinical Tropical Medicine, University of Heidelberg, Heidelberg, Germany

**Keywords:** alveolar echinococcosis, robotic surgery, major liver resection, DaVinci-assisted major liver resection, hemihepatectomy

## Abstract

We report a case of successful robot-assisted major liver resection in a patient with liver alveolar echinococcosis (AE). A 62-year-old male patient was incidentally diagnosed with a large infiltrative lesion in the right liver lobe suspicious for AE. A radical surgical resection as a right-sided hemihepatectomy was indicated. The operation was carried out *via* a robotic-assisted procedure using the DaVinci Xi Surgical System. The tumor measured 12.4 × 8.8 cm and was successfully resected through a suprapubic incision of 13 cm. The patient was free of pain after the second post-operative day. A fluid collection near the resection plate was easily drained without bile leakage. The patient had no surgical complications. Radical resection is inevitable for adequate curative therapy of AE and provides clear margins. Robotic surgery is a relatively new and safe option for curative resection of AE lesions, with remarkable advantages for patients and surgeons.

## Introduction

Alveolar echinococcosis (AE), caused by *Echinococcus (E.) multilocularis*, is widely distributed in the northern hemisphere. Other species are *E. granulosus* leading to cystic echinococcosis, and *E. vogeli* and *E. oligarthrus*, causing polycystic echinococcosis (1).

In Europe, the prevalence of AE is low and is estimated at 0.64/100,000 in Germany. Most infections exclusively affect the liver. In patients with very large liver masses and invasion of major hepatic vessels metastases to the lung and less frequently to the brain may occur. There are two treatment options: (1) parasitostatic treatment with benzimidazoles, which is indicated in cases where curative resection is not possible or suitable, and (2) radical resection with safety margins of 2 cm. However, safety margins of 1 mm have also been tolerated without recurrence of disease (2–5). Following an R0 resection, albendazole treatment is recommended for at least 2 years. Patients have to be followed for 10 years after surgical treatment. We report here in detail a case of robot-assisted major liver resection for alveolar echinococcosis treatment.

## Case Description

A 62-year-old male patient, incidentally diagnosed with a large infiltrative lesion in the liver during trauma clarification, presented himself in our hospital. An abdominal CT scan was performed to rule out trauma injuries and showed a poorly defined lesion in the right liver lobe suspicious for cholangiocellular carcinoma or alveolar echinococcosis (AE). The patient denied any abdominal or B symptoms, and there was no family history of malignant disease. He lived in a rural area in Germany.

**Figure 1:**
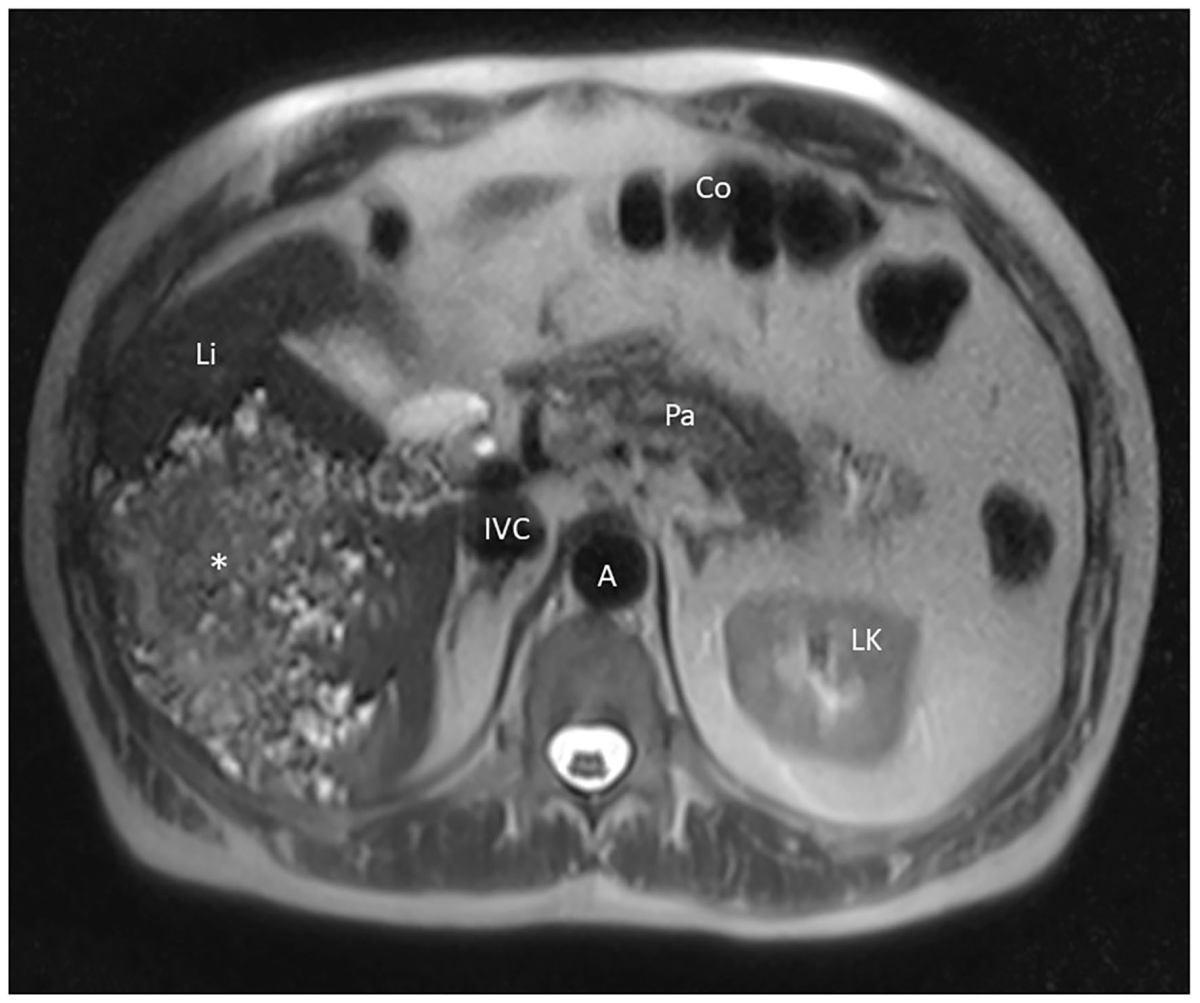
MRI imaging showing the AE lesion of our patient in the axial plane. Li, liver; Co, colon; Pa, pancreas; LK, left kidney; A, aorta; IVC, inferior vena cava, *AE tumor.

For further clarification, blood tests and an MRI scan of the abdomen were performed. Echinococcus serology was positive (*E. species* 1.541 optical density with a cut-off value of 0.988 and *E. multilocularis* 1.666 optical density with a cut-off value of 0.625). Immunoblot revealed 7-, 16-, 18-, and 26–28 kDa proteins in size. Blood tumor markers were in the normal range (CEA: 0.7 ug/l, CA 19–9: 23.4 U/ml, and AFP: 2.1 IU/ml). MRI imaging showed an enormous manifestation of AE in the right liver with microcystic and necrotic components that contacted the right pedicle (Figure 1); no further suspicious lesions were present in the liver or lymph nodes. There were no distant metastases (head, chest). Our multidisciplinary liver board discussed the case and recommended radical surgical resection of the lesion with a right-sided hemihepatectomy and parasitostatic treatment with albendazole.

**Figure 2:**
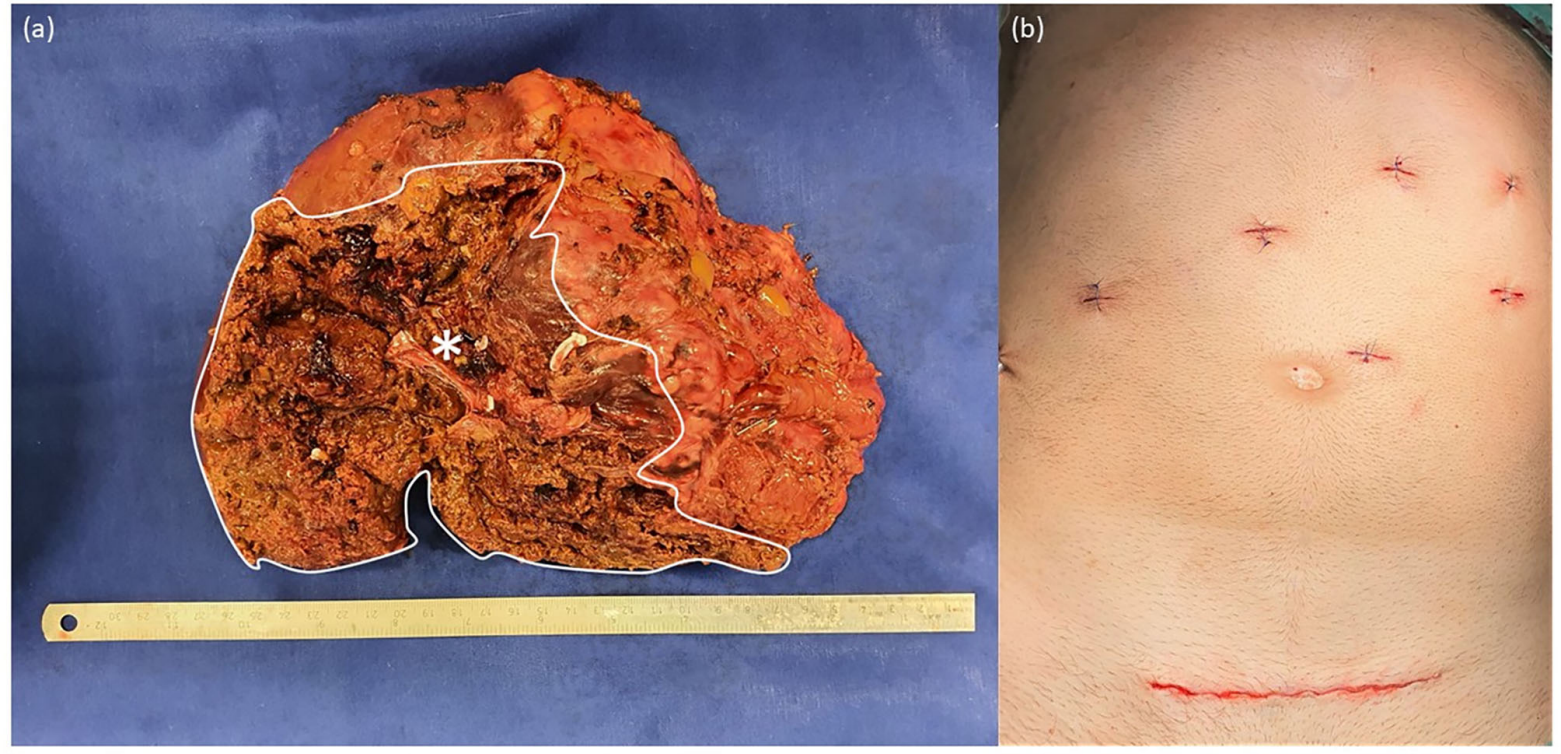
**(a)** Right hemi-liver including the tumor measuring 12.4 × 8.8 cm as well as segments 5, 6, 7, and 8. *AE tumor. **(b)** Postoperative scars after DaVinci-assisted right hemihepatectomy.

**Figure 3:**
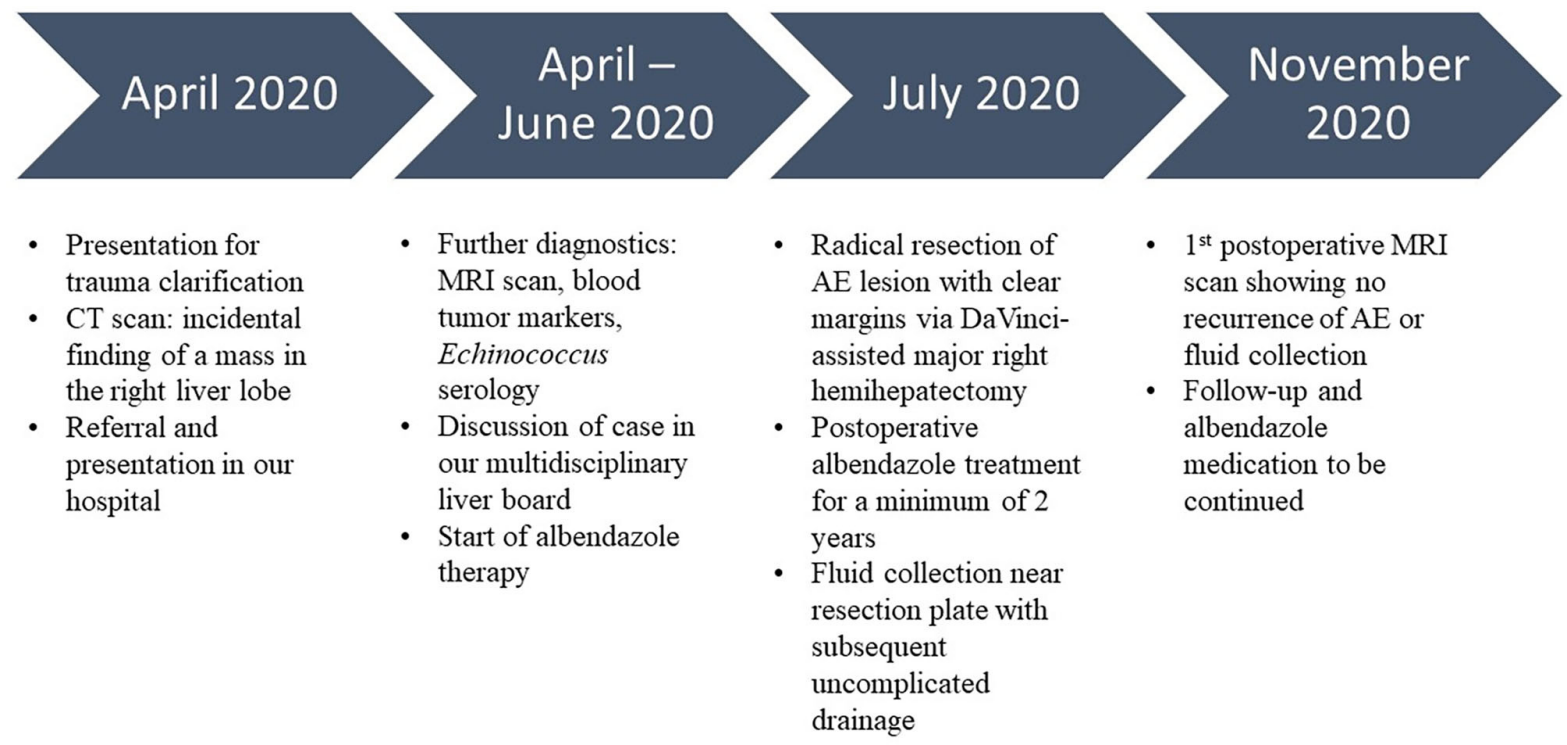
Timeline of the presented case.

The operation was carried out as a robotic-assisted procedure using the DaVinci Xi Surgical System (Intuitive Surgical Inc., United States). The tumor measured 12.4 × 8.8 cm (Figure 2a) and was successfully removed from the abdomen through a suprapubic incision of 13 cm (Figure 2b). Histopathological examinations revealed clear resection margins. The patient was mobile on the first post-operative day and was free of pain after the second post-operative day. Postoperatively, a fluid collection was observed near the resection plate that was easily drained without bile leakage. The patient had no surgical complications such as posthepatectomy biliary leakages or bleeding. Albendazole therapy was prescribed for 2 years after surgery, and follow-up examinations were scheduled. During the first 6 months of follow-up, the MRI scan showed no recurrent AE or fluid collection at the resection plate, and *E. species* serology was negative. The patient presented in a good general state of health and reported good recovery and return to daily activities and work. The timeline of the described case is summarized in Figure 3.

## Discussion

For successful treatment of AE, an expert consensus recommends a multidisciplinary discussion to plan the therapeutic strategy according to the stage of the disease. Preoperative treatment with parasitocidal agents is not routinely suggested in management of AE. However, it has been suggested to stabilize the progression of the disease with parasitocidal agents, which was administered in our patient (6). Radical surgery should be the treatment of choice in all patients where feasible and carried out in line with oncological standards leaving tumor-free margins of 2 cm. However, margins of 1 mm have also been tolerated without recurrence of the disease. Postoperatively, drug treatment with the benzimidazoles, an anthelmintic agent, for at least 2 years in R0-resection patients, or even for life in the case of incomplete resection, is recommended. The drug is usually administered at a daily dose of 800 mg, divided into 2 doses. After surgery and the start of benzimidazole therapy, e.g., with albendazole, surveillance for 10 years to detect possible recurrence is strongly advised. In palliative situations, interventional procedures are to be favored over palliative surgery, which is to be avoided in any situation possible (2–5).

Minimally invasive surgical procedures can improve post-operative mobilization and reduce wound-healing impairment compared with open surgery (7). Robotic-assisted surgery is widely used as a replacement for conventional laparoscopic surgery because of its enhanced precision (8), dexterity, and intuitiveness (9, 10). The crucial benefits of robotic surgery are lower overall morbidity rates and a shorter hospital stay than open surgery (10–13). Reduced post-operative pain (10, 12) and better cosmetic results (11) also improve patients' outcomes after robotic surgery. Robotic assistance systems, such as DaVinci Xi, can help overcome the disadvantages of conventional laparoscopic surgery by giving surgeons a better view of the operating field (high-definition, 3-dimensional vision with adjustable magnification) and by stabilizing the surgical instruments with an extended range of motion (7, 10, 14, 15). Additionally, the operating position is more comfortable for the surgeons, preventing fatigue, improving concentration, and reducing stress (10, 16). Despite scarce evidence for robotic liver surgery (7), liver resection safety and feasibility by robotic-assisted procedures have been well-demonstrated (8, 13–15). AE is not a malignant disease but needs to be surgically treated as such. The robotic system offered AE patients a safe tumor resection with clear margins, early post-operative mobilization, pain-free recovery, and optimal cosmetic results and provided surgeons with a stress-free and comfortable operating environment.

In the literature, several cases have been reported to undergo robot-assisted treatment of hepatic echinococcosis. Of these, most reports have focused on the approach to patients with cystic echinococcosis, which its management differs from patients with AE. In these patients, total cystectomy, minor, and major liver resections have been mostly utilized, as reported by Magistri et al. (17) and Felsenreich et al. (18). Conversely, few patients with AE have been reported to undergo liver resection using robotic approaches, especially major liver resection. Although Efanov have addressed the safety and feasibility of robot-assisted liver resection in a patient with AE, a bisegmentectomy was indicated for the patient rather than a major liver resection (19). Zhao et al. reported 5 cases, of which only 1 patient presents with AE; all other patients are diagnosed with cystic echinococcosis (20). This single case with AE underwent a right hemihepatectomy. Therefore, our report is one of the very first reports of robotic-assisted major liver resection for AE treatment. Of course, there are limitations with robotic surgery for AE, such as abdominal adhesions or lack of surgical experience, and careful evaluation of each case is necessary.

Rupture is a rare complication in AE and has only been described once in the literature due to trauma (21). The ruptured lesion was managed via repeated resections and albendazole medication. Although the intraoperative rupture of hepatic AE has not been reported, precautions should be taken during the operation and the resection has to be performed like a malignant tumor. For major hepatectomy, subcostal, mid-line, or suprapubic incisions can be performed for the safe removal of the tumor. Since subcostal incisions have been associated with a higher incidence of post-operative pulmonary complications and mid-line incisions carry a higher risk of developing post-operative incision hernia (22), a suprapubic incision seemed to be the best option. Additionally, suprapubic incisions lead to a better cosmetic result (23–25).

## Conclusion

In conclusion, AE is a rare disease that should be considered a differential diagnosis in patients with benign and malignant liver tumors. For adequate curative therapy, AE requires radical resection with clear margins and post-operative anti-parasitic drug treatment. Robotic surgery is a relatively new but safe option for curative resection of AE lesions with benefits for patients and surgeons.

## Data Availability Statement

The raw data supporting the conclusions of this article will be made available by the authors, without undue reservation.

## Ethics Statement

Ethical review and approval was not required for the study on human participants in accordance with the local legislation and institutional requirements. The patients/participants provided their written informed consent to participate in this study. Written informed consent was obtained from the individual(s) for the publication of any potentially identifiable images or data included in this article.

## Author Contributions

MG, VF, AR, and AMa prepared and wrote this article. MG, AR, and AMe were involved in managing the patient besides preparing the intraoperative pictures. MG, YK, MS, and AMa wrote and revised the manuscript as well as acted as the corresponding authors. AMe was the main surgeon and involved directly in managing the patient. All authors contributed to the article and approved the submitted version.

## Conflict of Interest

The authors declare that the research was conducted in the absence of any commercial or financial relationships that could be construed as a potential conflict of interest.

## Abbreviations

AE: alveolar echinococcosis
E: Echinococcus
MRI: magnetic resonance imaging
Li: liver
Co: colon
Pa: pancreas
LK: left kidney
A: aorta
IVC: inferior vena cava.
